# Association of Early Minocycline Use with Secondary Infections in Patients with Severe Fever with Thrombocytopenia Syndrome: A Multicenter Retrospective Cohort Study

**DOI:** 10.3390/pathogens15101032

**Published:** 2026-10-01

**Authors:** Yuanyuan Zhang, Yuan Jiang, Xuya Yuan, Jingyi Wu, Ting Wu, Jun Cheng, Yuyao Li, Lingling He, Jiankang Zhang, Chun Zhang, Zhiping Pan, Mengyu Liu, Xuejiao Ma, Jihua Xue, Ying Ye, Jiabin Li, Lifen Hu

**Affiliations:** 1 Department of Infectious Diseases, The First Affiliated Hospital of Anhui Medical University, Hefei 230022, China; 2 Anhui Province Key Laboratory of Infectious Diseases, Anhui Medical University, Hefei 230022, China; 3 Department of Infectious Diseases, Lu’an People’s Hosital, Lu’an 237011, China; 4 Department of Infectious Diseases, Chaohu Hospital of Anhui Medical University, Chaohu 238000, China; 5 Department of Infectious Diseases, Anqing Municipal Hospital, Anqing 246004, China

**Keywords:** severe fever with thrombocytopenia syndrome, minocycline, secondary infection, cytokines

## Abstract

Severe fever with thrombocytopenia syndrome (SFTS) is an emerging tick-borne infection disease in which secondary infections may worsen clinical outcomes, particularly in severe cases. This multicenter retrospective cohort study included 1975 laboratory-confirmed SFTS patients from three hospitals in Anhui, China (2011–2025), evaluating whether early minocycline use was associated with lower odds of secondary infections and differences in inflammatory cytokine levels. Propensity score matching was used to balance measured baseline characteristics between the minocycline and control groups. Logistic regression and exploratory subgroup analyses were performed. Among 1975 patients with confirmed SFTS, 548 received minocycline and 1427 did not. The incidence of secondary infection was lower in the minocycline group than in the control group (44.22% vs. 52.99%). Site-specific analysis showed that pulmonary infections were less frequent in the minocycline group (41.83% vs. 49.82%), whereas differences in bloodstream and urinary tract infections were not statistically significant. Multivariate logistic regression showed that early minocycline use was associated with lower odds of secondary infection (OR = 0.62, 95% CI: 0.47–0.82). In an exploratory cytokine analysis, serum interleukin-8 (IL-8) and IL-10 levels were lower in the minocycline group after matching. Early minocycline use was associated with lower odds of secondary infections, particularly pulmonary infections, in patients with SFTS.

## 1. Introduction

Severe fever with thrombocytopenia syndrome (SFTS) is a newly emerging infectious disease first reported in China in 2010 [1]. Its causative agent, initially named severe fever with thrombocytopenia syndrome virus (SFTSV), has since been renamed Dabie bandavirus, within the class *Bunyaviricetes*, order *Hareavirales*, family *Phenuiviridae*, genus *Bandavirus*, and species *Bandavirus dabieense* [2]. Over the past decade, both the reported incidence and geographic distribution of SFTS have expanded substantially. Endemic regions, previously limited to China, Japan, and South Korea, have now extended to Vietnam, Myanmar, Thailand, and Pakistan, underscoring a growing public health threat across Asia [3]. Between 2011 and 2021, a total of 18,902 confirmed cases were documented in China alone, with a persistently upward incidence trajectory and pronounced spatiotemporal clustering [4].

Clinically, SFTS is characterized by high fever, leukopenia, thrombocytopenia, gastrointestinal symptoms, lymphadenopathy, and elevated serum enzymes. Severe cases may progress rapidly to hemorrhagic manifestations, neurological complications, shock, disseminated intravascular coagulation, multiple organ dysfunction syndrome, and death [5]. Despite the substantial disease burden, no vaccine or specific antiviral agent has been formally approved for SFTS. Current treatment remains mainly supportive and includes antiviral therapy, immunomodulation, organ support, and aggressive management of complications [6]. Secondary infections are clinically important complications in patients with SFTS, especially in severe and critically ill cases [7]. Pulmonary infection is particularly common and may contribute to respiratory failure, prolonged hospitalization, and poor prognosis. Bloodstream infection and urinary tract infection may also occur during hospitalization, especially in patients requiring intensive care, invasive procedures, or broad-spectrum antimicrobial exposure [8]. The bacterial pathogens are dominated by Gram-negative bacilli, particularly *Klebsiella pneumoniae* and *Acinetobacter baumannii*, whereas fungi are represented mainly by *Aspergillus fumigatus* and *Candida albicans* [9]. Consequently, early recognition and appropriate management of concurrent infections constitute an essential component of comprehensive SFTS care.

Minocycline, a broad-spectrum tetracycline antibiotic, possesses not only potent antibacterial activity but also well-documented anti-inflammatory, immunomodulatory, and antiviral properties [10]. Its high lipophilicity and favorable tissue penetration enable substantial accumulation within macrophages, a property that may facilitate the elimination of intracellular pathogens [11]. The Centers for Disease Control and Prevention (CDC) guidelines recommend tetracyclines, specifically doxycycline, as first-line therapy for suspected or confirmed tick-borne rickettsial infections; minocycline may be considered as an alternative [12]. Because SFTS is primarily transmitted by tick bites, and ticks can carry multiple pathogens concurrently, including Rickettsia, Mycoplasma, and Ehrlichia species, tetracyclines may provide additional clinical value in patients with suspected bacterial co-infections [13]. Beyond its antimicrobial effects, minocycline exerts several non-antibiotic pharmacological actions, including inhibition of pro-inflammatory cytokine release, modulation of the nuclear factor kappa B (NF-κB) and mitogen-activated protein kinase (MAPK) signaling pathways, reduction in matrix metalloproteinase activity, and regulation of macrophage and microglial activation [14,15]. In other viral infections, including dengue virus, respiratory syncytial virus, and Japanese encephalitis virus infections, minocycline has also been reported to exert antiviral or neuroprotective effects [16,17,18].

However, direct evidence regarding the role of minocycline in SFTS remains limited. It remains unclear whether minocycline use is associated with a reduced risk of secondary infection or altered inflammatory cytokine profiles in patients with SFTS. Therefore, this multicenter retrospective cohort study aimed to evaluate the association between early minocycline use and secondary infections in patients with SFTS using propensity score matching. We also explored its association with serum inflammatory cytokine.

## 2. Materials and Methods

### 2.1. Study Design and Population

**Figure 1:**
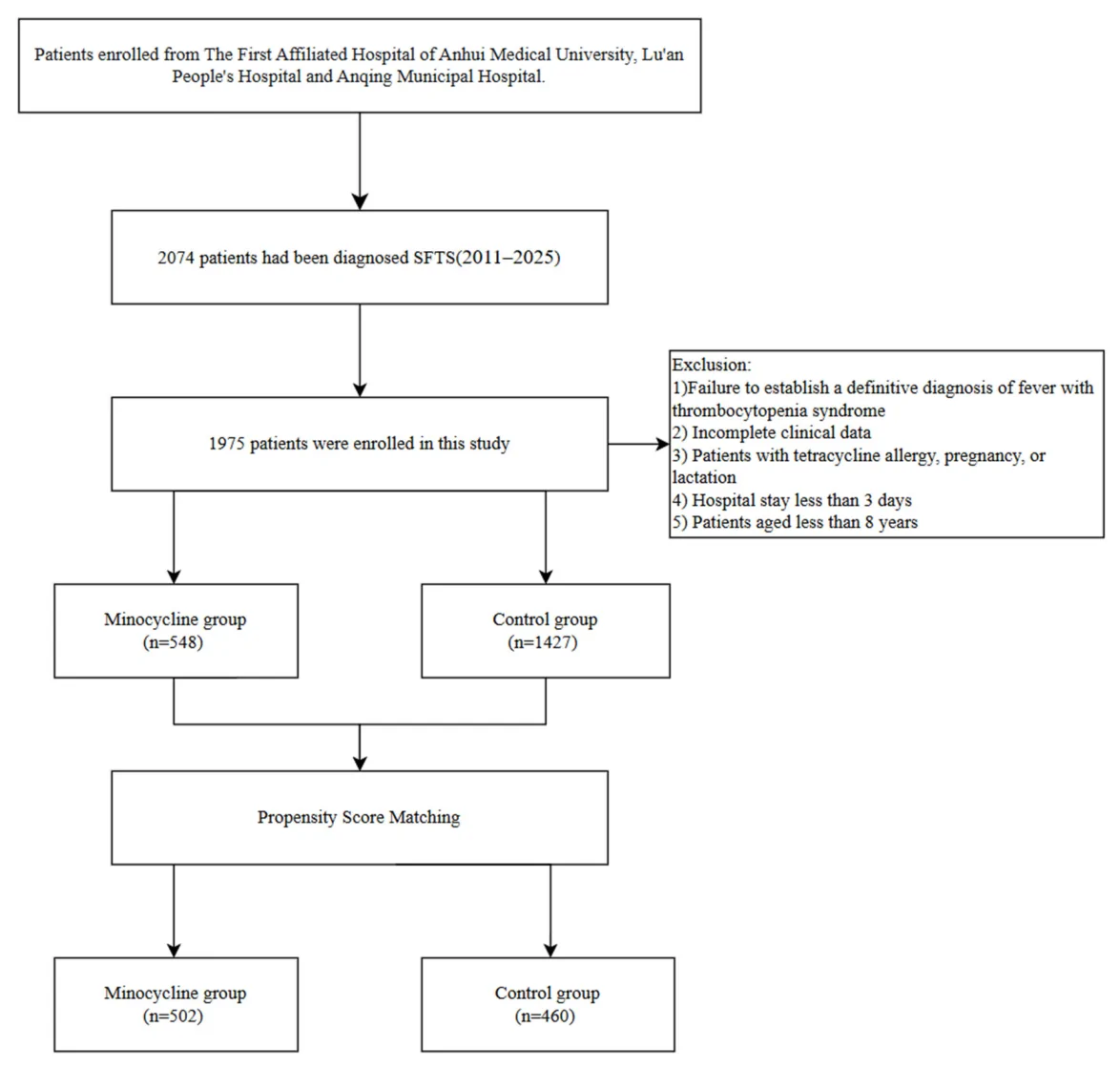
Flowchart of patient recruitment and exclusion criteria. Abbreviations: SFTS, severe fever with thrombocytopenia syndrome.

This was a multicenter retrospective cohort study. Data were collected from laboratory-confirmed SFTS patients at three hospitals in Anhui Province between January 2011 and December 2025 (Figure 1). These hospitals include the First Affiliated Hospital of Anhui Medical University, Lu’an People’s Hospital, and Anqing Municipal Hospital.

Patients were eligible for inclusion if they met the diagnostic criteria specified in the Guidelines for the Prevention and Treatment of Severe Fever with Thrombocytopenia Syndrome [19]. Laboratory-confirmed SFTS required evidence of active or recent SFTSV infection, defined as at least one of the following: positive SFTSV nucleic acid test; positive SFTSV-specific IgM antibody; seroconversion; or a fourfold or greater rise in IgG antibody titer between acute- and convalescent-phase sera. Isolated IgG positivity was not considered evidence of active infection and was not accepted as an inclusion criterion. The exclusion criteria were as follows: uncertain diagnosis of SFTS; missing key clinical or outcome data; known tetracycline allergy, pregnancy, or lactation; hospitalization duration of less than 3 days; and age younger than 8 years.

This study protocol has been reviewed and approved by the Ethics Committee of Anhui Medical University (No. 20200980) and conducted in accordance with the Declaration of Helsinki. The requirement for informed consent was waived because of the retrospective nature of the study.

### 2.2. Definitions and Data Collection

Patients were classified according to early minocycline exposure. Minocycline was initiated on the day of hospital admission in all patients assigned to the minocycline group and administered for three consecutive days. Minocycline was generally administered at a dose of 100 mg every 12 h, with a loading dose at the first administration when clinically indicated. The specific patient-level rationale for prescribing or withholding minocycline was not systematically available in the retrospective medical records. In contrast, the control group did not receive prophylactic antibiotics during the first three days of hospitalization.

Secondary infection was defined as a new infection arising after 48 h of hospitalization according to clinical, radiological, laboratory, and microbiological criteria. Pulmonary infection was diagnosed based on new or progressive pulmonary infiltrates on chest imaging plus compatible clinical manifestations and laboratory evidence, including cough, sputum production, dyspnea, hypoxemia, fever or recurrent fever, elevated inflammatory markers, and microbiological evidence when available [20]. Bloodstream infection was diagnosed on the basis of compatible clinical manifestations and pathogen detection from blood culture or blood-based next-generation sequencing [21]. Urinary tract infection was diagnosed on the basis of compatible urinary or systemic manifestations together with urinalysis and urine culture when available. Microbiological testing was performed according to clinical indication and was not standardized across all patients.

Since no universally accepted criteria exist for identifying severe SFTS, we defined disease severity by integrating established clinical presentations and serious complications as outlined in the consensus guidelines [19]. Specifically, patients were classified as having severe disease if they developed any of the following conditions within 72 h after hospitalization: (1) failure or dysfunction of major organs, including respiratory, cardiac, or renal systems, as well as DIC or viral encephalitis; (2) marked central nervous system involvement, manifested as coma, delirium, or repeated seizures; or (3) clinically significant bleeding events involving the central nervous system, gastrointestinal tract, pulmonary system, or uterine cavity.

Demographic characteristics, comorbidities, clinical manifestations, laboratory results, microbiological findings, imaging results, treatment measures, length of hospital stay, and clinical outcomes were collected from electronic medical records. Demographic variables included age, sex, systolic blood pressure, and diastolic blood pressure. Comorbidities included hypertension, diabetes, and other underlying diseases. Laboratory parameters were selected based on their clinical relevance to SFTS severity and secondary infection risk. Hematological variables were used to assess leukopenia, lymphopenia, thrombocytopenia, and anemia; biochemical variables were used to evaluate hepatic injury, renal function, nutritional status, and metabolic abnormalities; coagulation variables were used to assess coagulation dysfunction; inflammatory markers were used to characterize systemic inflammatory response; and viral load was used as an indicator of viral burden. These parameters were included to characterize baseline severity and to adjust for potential confounding in the association between minocycline exposure and secondary infection. Laboratory parameters included albumin, globulin, total bilirubin, alanine aminotransferase, aspartate aminotransferase, alkaline phosphatase, gamma-glutamyl transpeptidase, lactate dehydrogenase, urea, creatinine, blood glucose, C-reactive protein, procalcitonin, white blood cell count, neutrophil count, lymphocyte count, hemoglobin, platelet count, prothrombin time, international normalized ratio, activated partial thromboplastin time, fibrinogen, D-dimer, and SFTSV viral load. Serum cytokines, including nterleukin-1 beta (IL-1β), IL-2, IL-4, IL-5, IL-6, IL-8, IL-10, IL-12p70, IL-17, tumor necrosis factor-alpha (TNF-α), interferon-alpha (IFN-α), and interferon-gamma (IFN-γ), were collected when available. Imaging studies included chest computed tomography. Major therapeutic interventions, including intravenous immunoglobulin, glucocorticoids, antiviral therapy, and other supportive treatments, were also recorded.

### 2.3. Outcome Measures and Follow-Up

The outcome was 28-day all-cause mortality after admission. Survival status was obtained from hospital records. For patients discharged unexpectedly because of clinical deterioration, survival status and date of death were confirmed by telephone follow-up. Patients who survived beyond 28 days were censored at day 28.

### 2.4. Propensity Score Matching

Propensity score matching was performed to reduce selection bias and baseline confounding between the minocycline and control groups [22]. Variables included in the propensity score model were selected based on clinical relevance and baseline imbalance and included age, sex, comorbidities, vital signs, key laboratory parameters, SFTSV viral load, and major indicators of disease severity. A 1:1 nearest-neighbor matching method was applied with a caliper of 0.1. Covariate balance before and after matching was assessed using standardized mean differences. An absolute standardized mean difference (SMD) less than 0.10 was considered to indicate acceptable balance.

### 2.5. Statistical Analysis

Continuous variables with a normal distribution were presented as mean and standard deviation and compared using the independent-samples *t* test. Continuous variables with a non-normal distribution were presented as median and interquartile range and compared using the Mann–Whitney U test or Kruskal–Wallis test, as appropriate. Categorical variables were presented as counts and percentages and compared using the chi-square test or Fisher’s exact test.

Univariate logistic regression was used to identify factors associated with secondary infection. Variables with clinical relevance and variables associated with secondary infection in univariate analysis were further included in multivariate logistic regression models. Results were reported as odds ratios, adjusted odds ratios, 95% confidence intervals, and *p*-value.

Subgroup analyses were performed according to sex, age, viral load, diabetes status, intravenous immunoglobulin use, and glucocorticoid use. The age cutoff was determined exploratorily using receiver operating characteristic curves and the Youden index, whereas the viral-load threshold of 10^7^ copies/mL was based on previous SFTS studies [23]. Interaction terms were used to assess whether the association between minocycline use and secondary infection differed across subgroups. Subgroup analyses were considered exploratory. A two-sided *p*-value < 0.05 was considered statistically significant. Statistical analyses were performed using R version 3.6.3 and GraphPad Prism version 9.

## 3. Results

### 3.1. Baseline Characteristics of Study Participants

A total of 98 patients were excluded because their hospital stay was less than 3 days, including 81 who died during hospitalization and 17 who were transferred to other hospitals, and compared them with those of patients included in the primary analysis cohort(Supplementary Table S1). After these exclusions, a total of 1975 patients with laboratory-confirmed SFTS were included in this study. Among them, 548 patients received minocycline and 1427 patients did not. Before propensity score matching, differences were observed between the two groups in several baseline variables, including age, albumin, total bilirubin, alanine aminotransferase, aspartate aminotransferase, lactate dehydrogenase, high-sensitivity C-reactive protein, and D-dimer, indicating baseline imbalance between the groups. No statistically significant differences were observed in sex, globulin, blood urea nitrogen, and several other indicators (Table 1).

After propensity score matching, each group contained 502 patients. The baseline characteristics of the minocycline and control groups were generally balanced and comparable, with all SMDs < 0.10 (Figure S1). The 28-day mortality rate was lower in the minocycline group than in the control group after propensity score matching. However, the difference was not statistically significant (14.5% vs. 16.5%; *p* = 0.411).

### 3.2. Association Between Minocycline Use and Secondary Infection in SFTS Patients

**Figure 2:**
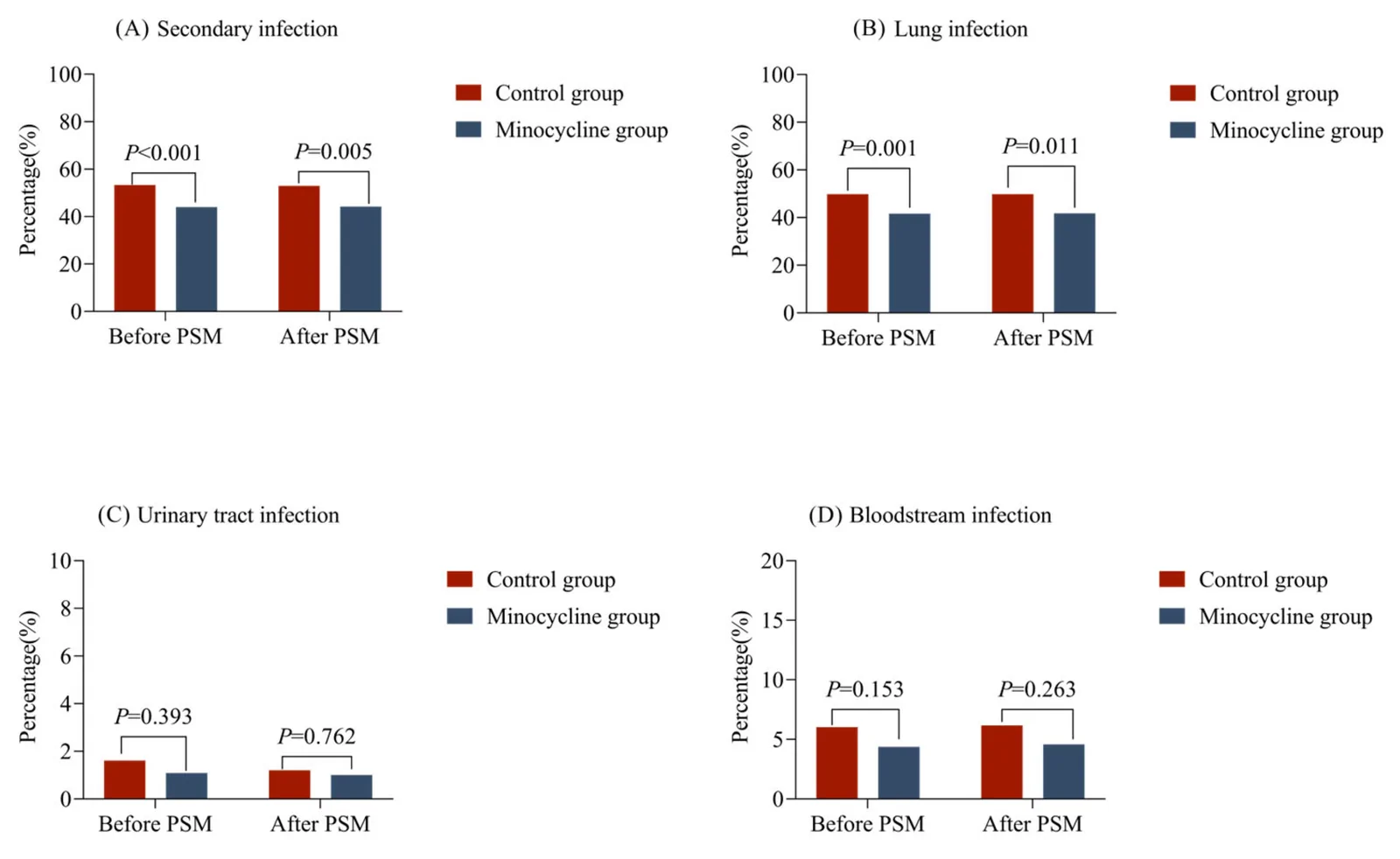
The incidence of infection in the control and minocycline groups before and after propensity score matching. (**A**) Incidence of secondary infection in the minocycline and control groups. (**B**) Incidence of lung infection in the minocycline and control groups. (**C**) Incidence of urinary tract infection in the minocycline and control groups. (**D**) Incidence of bloodstream infection in the minocycline and control groups. Abbreviations: PSM, propensity score matching.

After propensity score matching, the incidence of secondary infection was significantly lower in the minocycline group than in the control group (44.22% vs. 52.99%; *p* = 0.005). Site-specific analysis showed that pulmonary infection occurred less frequently in the minocycline group than in the control group (41.83% vs. 49.82%; *p* = 0.011) (Figure 2). The incidence rates of bloodstream infection and urinary tract infection were numerically lower in the minocycline group, but the differences were not statistically significant.

Minocycline use was associated with a lower incidence of secondary infection in several subgroups, including patients aged 60 years or younger, female patients, patients with viral load of 10^7^ copies/mL or lower, patients without diabetes, and patients who did not receive glucocorticoids or intravenous immunoglobulin (Figures S2–S7). The association was most apparent for pulmonary infection. However, the associations between minocycline use and bloodstream or urinary tract infection remained uncertain.

### 3.3. Pathogen Spectrum of SFTS Patients with Secondary Infection

**Figure 3:**
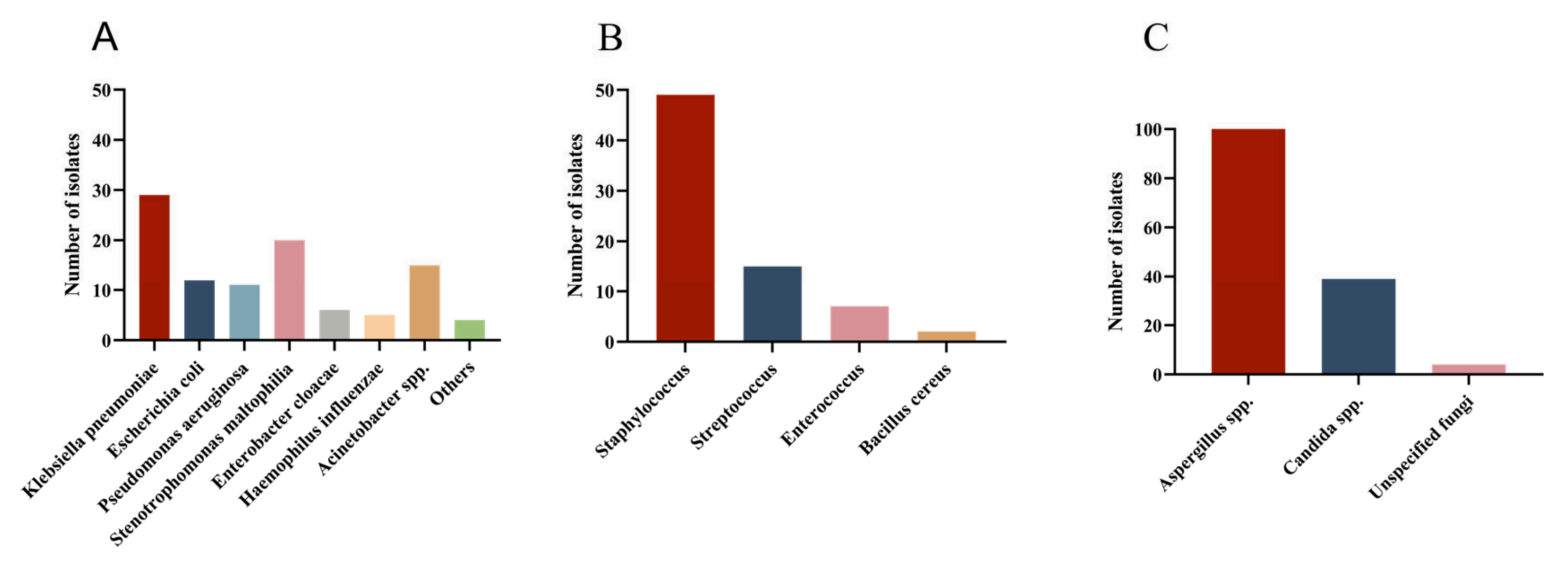
Microbiology among patients with secondary infection and available pathogen results. (**A**) Distribution of Gram-negative bacteria. (**B**) Distribution of Gram-positive bacteria. (**C**) Distribution of fungi.

Among the patients diagnosed with secondary infection, a total of 319 bacterial and fungal pathogen isolates were identified. As shown in Figure 3, 102 (32.0%) isolates were Gram-negative bacilli, including 29 *Klebsiella pneumoniae*, 20 *Stenotrophomonas maltophilia*, 15 *Acinetobacter* spp., 12 *Escherichia coli*, 11 *Pseudomonas aeruginosa*, 6 *Enterobacter cloacae*, 5 *Haemophilus influenzae*, and 1 isolate each of *Raoultella ornithinolytica*, *Ralstonia* spp., *Cupriavidus gilardii*, and *Aeromonas hydrophila*. We also detected 73 (22.9%) isolates of Gram-positive bacteria, comprising 49 *Staphylococcus* spp., 15 *Streptococcus* spp., 7 *Enterococcus* spp. and 2 *Bacillus cereus*. Additionally, 144 (45.1%) fungal isolates were identified, including 101 *Aspergillus* spp., 39 *Candida* spp., and 4 unspecified fungi. In addition, 7 patients were diagnosed with *Mycoplasma pneumoniae* infection. Notably, 3 patients without a history of tuberculosis were diagnosed with active *Mycobacterium tuberculosis* infection.

### 3.4. Logistic Regression Analysis of Factors Associated with Secondary Infection in SFTS Patients

Univariate logistic regression analysis showed that minocycline use, age, serum albumin, total bilirubin, aspartate aminotransferase, lactate dehydrogenase, serum creatinine, blood glucose, C-reactive protein, lymphocyte count, hemoglobin, platelet count, activated partial thromboplastin time, intravenous immunoglobulin use, and glucocorticoid use were associated with secondary infection.

**Figure 4:**
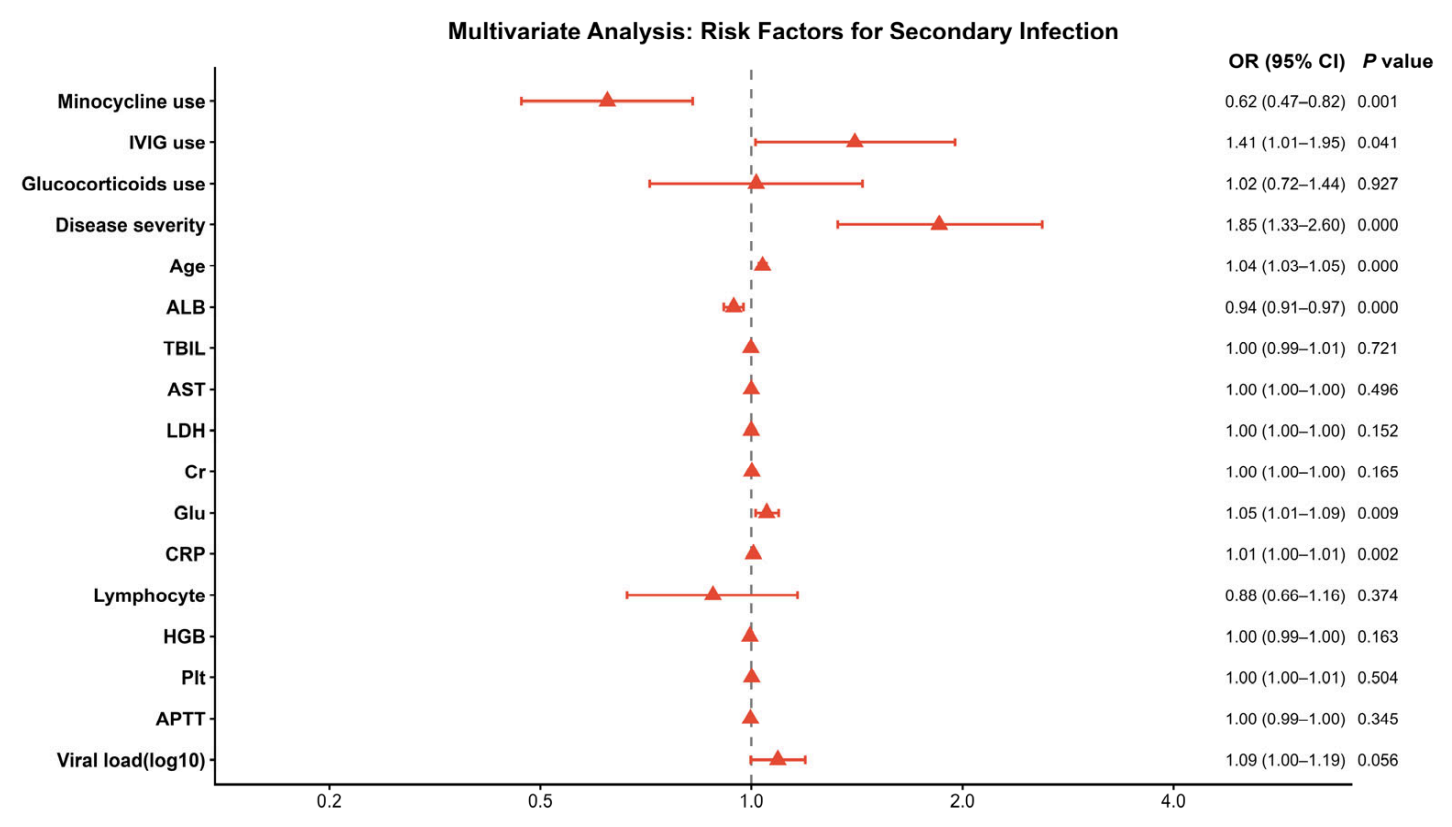
Multivariate logistic regression analysis of factors associated with combined infection in SFTS patients after PSM. Abbreviations: ALB, albumin; TBIL, total bilirubin; AST, aspartate aminotransferase; LDH, lactate dehydrogenase; Cr, creatinine; GLU, glucose; CRP, C-reactive protein; HGB, hemoglobin; Plt, platelet count; APTT, activated partial thromboplastin time; PSM, propensity score matching.

Variables with clinical relevance and statistical significance in univariate analysis were included in the multivariate logistic regression model (Figure 4). Early minocycline use was independently associated with a lower risk of secondary infection (OR = 0.62, 95% CI: 0.47–0.82, *p* = 0.001), corresponding to odds approximately 38% lower compared with the control group.

Additionally, both IVIG use and disease severity (OR = 1.85, 95% CI: 1.33–2.60, *p* < 0.001) were independently associated with increased odds of secondary infection. For each 1-year increase in age, the odds of secondary infection increased by approximately 4% (OR = 1.04, 95% CI: 1.03–1.05, *p* < 0.001). Higher albumin levels were associated with a lower odds of secondary infection; for each 1 g/L increase in albumin, the odds decreased by approximately 6% (OR = 0.94, 95% CI: 0.91–0.97, *p* < 0.001). Higher blood glucose levels were associated with increased odds of secondary infection; for each 1 mmol/L increase in blood glucose, the odds increased by approximately 5% (OR = 1.05, 95% CI: 1.01–1.09, *p* = 0.009). Higher C-reactive protein levels were also associated with increased odds of secondary infection (OR = 1.01, 95% CI: 1.00–1.01, *p* = 0.002).

### 3.5. Effect of Minocycline on the Risk of Infection in SFTS Patients from Different Populations

**Figure 5:**
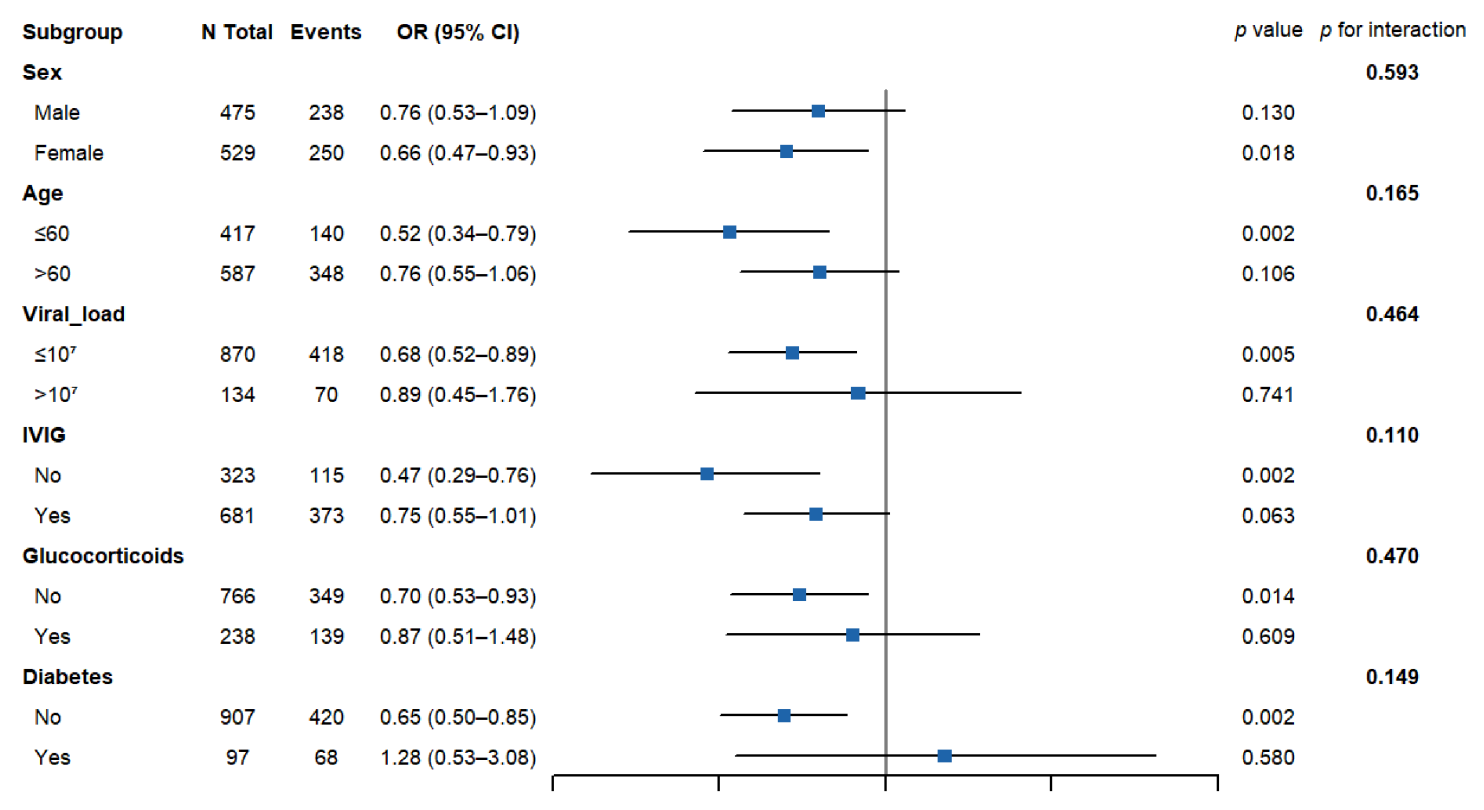
Stratified analyses of the associations between minocycline use and the incidence of combined infection. Abbreviations: CI, confidence interval; OR, odds ratio; IVIG, intravenous immunoglobulin.

To systematically evaluate the potential heterogeneity in the association between minocycline and the risk of secondary infection among patients with SFTS, we conducted subgroup analyses stratified by gender, age, concomitant use of intravenous immunoglobulin (IVIG), baseline viral load, glucocorticoids use, and the presence of diabetes mellitus (Figure 5). Interaction effects between minocycline treatment and each stratification variable were formally assessed using likelihood ratio tests (LRTs). Overall, minocycline treatment consistently demonstrated a trend toward a reduced risk of co-infection across most subgroups, and all interaction *p*-values exceeded 0.05, indicating that the protective association was not significantly modified by these baseline characteristics, thus supporting a robust and homogeneous treatment effect across diverse patient populations.

In the within-subgroup analyses, the odds ratios (ORs) for minocycline were below unity in the vast majority of strata, reinforcing its broad potential for clinical benefit. Statistically significant associations were observed in several subgroups: female patients (OR 0.66, 95% CI 0.47–0.93, *p* = 0.018), those aged ≤ 60 years (OR 0.56, 95% CI 0.34–0.79, *p* = 0.002), patients with viral load ≤ 10^7^ copies/mL (OR 0.68, 95% CI 0.52–0.89, *p* = 0.005), those not receiving IVIG (OR 0.47, 95% CI 0.29–0.76, *p* = 0.002), patients not treated with glucocorticoids (OR 0.70, 95% CI 0.53–0.93, *p* = 0.014), and those without diabetes (OR 0.65, 95% CI 0.50–0.85, *p* = 0.002).

### 3.6. Association Between Minocycline Use and Serum Cytokines in SFTS Patients

A total of 737 patients with confirmed SFTS admitted between 2019 and 2025 had available cytokine data and were included in the cytokine analysis (Table 2). After matching for age, sex, underlying diseases, and viral load, 197 patients were included in the minocycline group and 197 in the control group. Baseline characteristics were generally comparable between the two groups after matching (Figure S8).

After matching, serum IL-8 and IL-10 levels were significantly lower in the minocycline group than in the control group (Table 3). IL-1β, TNF-α, and IFN-α levels showed decreasing trends in the minocycline group, but the differences were not statistically significant. Because cytokine data were available only for a subset of patients and were measured at a single time point, these findings should be interpreted as exploratory.

## 4. Discussion

In this multicenter retrospective cohort study of patients with laboratory-confirmed SFTS, early minocycline use was associated with a lower risk of secondary infection during hospitalization, particularly pulmonary infection. This association was consistent in the unmatched cohort, after propensity score matching, and in the multivariable model (OR = 0.62, 95% CI: 0.47–0.82), and it was driven predominantly by pulmonary infection, which accounted for the majority of secondary infections in our cohort. We did not detect statistically significant associations for bloodstream or urinary tract infection; given the small numbers of these events, these null findings are more likely to reflect limited power than a true absence of effect. Pulmonary infection accounted for the majority of secondary infections in our cohort, consistent with reports from endemic regions of China in which respiratory specimens yielded most isolates [9]. Beyond pulmonary infection, patients with SFTS may develop bloodstream infection, urinary tract infection, and, less commonly, catheter-related and intra-abdominal infections, particularly among those with prolonged hospitalization or invasive devices [21].

The observed association between minocycline use and lower pulmonary infection risk may be explained by several possible mechanisms. Minocycline has broad antibacterial activity, including activity against several atypical and intracellular pathogens [10,11]. Because SFTS is transmitted by tick bites and may coexist with other tick-borne bacterial infections, early tetracycline-class antibiotic exposure may be beneficial in selected patients with suspected bacterial co-infection [19,20]. Minocycline has favorable tissue penetration and can accumulate within inflammatory cells, which may support its antimicrobial effects in infected tissues. Garrido-Mesa et al. reported that minocycline exerts anti-inflammatory and immunomodulatory effects by inhibiting various enzymes and regulating immune-cell activation and proliferation [24]. Minocycline has also been shown to attenuate the PAR2-IL-8 axis in keratinocytes, thereby modulating pro-inflammatory responses in the skin [25]. In studies of COVID-19, Al-Kuraishy et al. reported that minocycline may reduce complications by alleviating cytokine storm, as reflected by decreased IL-6, IL-1, and TNF-α levels [26]. During inflammatory responses, macrophages, microglia, and other immune cells secrete large amounts of pro-inflammatory cytokines, including TNF-α, IL-1β, and IL-6 [27]. Minocycline can suppress the synthesis and release of pro-inflammatory cytokines such as TNF-α and IL-1β by inhibiting the NF-κB and MAPK signaling pathways [24,28]. During acute infection, IL-10 limits immune-response intensity and suppresses excessive inflammation, thereby protecting tissues from immune-mediated injury and promoting the resolution of inflammation after pathogen clearance [29]. Therefore, by attenuating upstream pro-inflammatory signals, minocycline may reduce the need for compensatory IL-10-mediated anti-inflammatory responses, resulting in the physiological downregulation of IL-10 secretion. This interpretation is consistent with the significantly lower IL-10 levels observed in the minocycline group in the present study. In addition to its immunomodulatory effects, minocycline has been reported to inhibit the replication of several RNA viruses, and these antiviral effects may be independent of interferon signaling [10]. Saha et al. found that minocycline inhibited influenza A virus replication across different viral strains and cell lines by targeting the MEK/ERK signaling pathway and blocking the nuclear export of viral ribonucleoproteins [30].

The cytokine findings in this study provide additional exploratory evidence supporting a potential immunomodulatory association. After matching, IL-8 and IL-10 levels were significantly lower in the minocycline group. IL-8 is a key chemokine involved in neutrophil recruitment and inflammatory cell infiltration, and elevated IL-8 levels have been associated with severe inflammatory responses in infectious diseases [31,32]. A lower IL-8 level in the minocycline group may suggest reduced inflammatory activation or tissue inflammatory recruitment. IL-10 is generally considered an anti-inflammatory cytokine, but excessive IL-10 elevation during viral infection may also reflect immune dysregulation, impaired antiviral responses, and disease severity [30,32]. Previous studies have suggested that IL-10 is associated with viral load, cytokine storm, organ injury, and poor prognosis in SFTS [33]. In the context of secondary infections, a prospective multicenter cohort study found that secondary bacterial infections occurred in 26.7% of steroid-treated SFTS patients, and inflammatory cytokines could not be effectively reduced by steroid or plasma exchange treatments [34]. Therefore, the lower IL-10 levels observed in the minocycline group may reflect a less severe immune-dysregulation state. Nevertheless, because cytokines were measured at a single time point and only in a subset of patients, these results should not be interpreted as proof that minocycline directly regulates IL-8 or IL-10 in SFTS.

The subgroup analyses suggested that the association between minocycline use and lower secondary infection risk was generally consistent across several patient subgroups. The findings suggest that minocycline may be considered as a potential adjunctive option in high risk of secondary pulmonary infection, or early signs of bacterial or fungal complications.

Although our study did not directly evaluate antimicrobial resistance, a systematic review and meta-analysis of randomized controlled trials on tetracycline post exposure prophylaxis (PEP) found that tetracycline PEP was associated with an increased prevalence of tetracycline resistance in Neisseria gonorrhoeae (pooled OR 2.3, 95% CI 0.9–3.4), although this did not reach statistical significance, and a significant increase in commensal Neisseria species (OR 2.9, 95% CI 1.5–5.5) [35]. In the SFTS population specifically, a retrospective study of 718 patients found that 38.7% developed bacterial co-infection, with multidrug-resistant bacteria accounting for 33.5% of all isolates, and multidrug-resistant infection significantly increased the risk of fatal outcomes (OR 1.736) [36]. While the tetracycline PEP data derive from sexually transmitted infections studies and not from SFTS patients, and our study did not systematically collect antimicrobial susceptibility data, these findings collectively highlight the need to weigh the potential benefits of minocycline against the risk of promoting antimicrobial resistance. Antimicrobial stewardship principles should guide any decision regarding minocycline use in SFTS patients, and future prospective studies with systematic resistance surveillance are warranted.

Several limitations should be acknowledged. First, this was a retrospective observational study, and residual or unmeasured confounding cannot be fully excluded despite propensity score matching and multivariate adjustment. In addition, exact patient-level dates of secondary infection diagnosis were not consistently available, preventing reliable calculation of the interval from admission to infection and precluding formal time-dependent or landmark analyses based on infection timing. Second, patients hospitalized for less than 3 days were excluded because they had a limited observation window for an outcome defined as infection occurring more than 48 h after admission. This exclusion may nevertheless have introduced selection bias if short-stay patients differed systematically from the analyzed cohort, particularly among patients with early transfer, discharge against medical advice, or rapid clinical deterioration. Third, the long multicenter study period encompassed changes in diagnostic capacity, antimicrobial practices, supportive care, and availability of next-generation sequencing, all of which may have affected exposure and outcome ascertainment. Fourth, detailed information on concomitant antimicrobial therapy was not uniformly available, leaving the possibility of residual treatment-related confounding. Fifth, cytokine analysis was restricted to a subset of patients and relied on single-time-point measurements. Finally, minocycline-related adverse reactions and the risk of selecting resistant organisms were not systematically assessed. Accordingly, strategy involving minocycline should be restricted to clearly defined high-risk populations and evaluated in prospective studies incorporating microbiological surveillance and resistance monitoring.

## 5. Conclusions

In this multicenter retrospective cohort study, early minocycline use was associated with lower odds of secondary infection in patients with SFTS, particularly pulmonary infection. Minocycline use was also associated with lower serum IL-8 and IL-10 levels in an exploratory cytokine-analysis cohort. Prospective multicenter studies with standardized exposure definitions, detailed antimicrobial-treatment data, pathogen profiles, time-to-infection analysis, and safety assessment are needed to clarify the clinical role of minocycline in SFTS.

## Figures and Tables

**Table 1:** Clinical and laboratory characteristics of the minocycline and control groups before and after PSM.

Characteristics	Control Group (*n* = 1427)	Minocycline Group (*n* = 548)	*p*-Value	SMD	Control Group (*n* = 502)	Minocycline Group (*n*= 502)	*p*-Value	SMD
Basic characteristic								
Age, year	68.00 (59.00, 74.00)	63.00 (53.75, 71.00)	<0.001	0.354	63.00 (54.00, 71.00)	63.00 (55.00, 72.00)	0.365	0.063
Male, *n* (%)	604 (42.3%)	257 (46.9%)	0.074	0.046	243 (48.4%)	232 (46.2%)	0.527	0.022
SBP, (mmHg)	115.00 (103.00, 127.00)	112.00 (99.00, 124.00)	0.001	0.159	110.50 (100.00, 123.00)	113.00 (100.00, 124.00)	0.384	0.025
DBP, (mmHg)	72.00 (64.00, 80.00)	70.00 (62.00, 78.00)	0.003	0.155	70.00 (63.00, 77.00)	70.00 (62.00, 78.00)	0.777	0.006
History								
DM, *n* (%)	204 (14.3%)	52 (9.5%)	0.006	0.048	46 (9.2%)	51 (10.2%)	0.669	0.010
Hypertension, *n* (%)	449 (31.5%)	130 (23.7%)	0.001	0.077	119 (23.7%)	123 (24.5%)	0.825	0.008
Laboratory parameters								
GLU, (mmol/L)	6.81 (5.61, 9.39)	7.32 (5.93, 9.62)	0.002	0.006	6.92 (5.70, 9.55)	7.30 (5.88, 9.53)	0.139	0.009
ALB, (g/L)	32.40 (28.70, 36.30)	30.20 (26.30, 34.42)	<0.001	0.355	30.50 (26.50, 34.70)	30.40 (26.60, 34.70)	0.802	0.010
GLO, (g/L)	26.30 (23.30, 29.85)	26.35 (23.30, 29.50)	0.900	0.057	26.20 (23.20, 29.67)	26.50 (23.42, 29.58)	0.594	0.004
TBIL, (umol/L)	11.47 (8.27, 18.67)	14.55 (9.70, 23.87)	<0.001	0.272	13.90 (9.01, 25.17)	14.10 (9.44, 22.86)	0.884	0.045
ALT, (U/L)	73.00 (41.00, 129.00)	85.00 (47.75, 155.00)	<0.001	0.161	87.00 (49.00, 143.00)	82.50 (45.00, 146.75)	0.465	0.004
AST, (U/L)	141.00 (73.50, 280.50)	167.00 (85.00, 343.50)	0.003	0.134	163.00 (81.00, 345.50)	160.50 (82.00, 331.75)	0.622	0.050
LDH, (U/L)	560.00 (360.35, 992.00)	889.50 (451.00, 1824.25)	<0.001	0.381	691.00 (373.75, 1428.00)	803.50 (428.75, 1733.75)	0.006	0.086
BUN, (mmol/L)	6.57 (4.94, 9.30)	6.31 (4.88, 8.80)	0.290	0.066	6.38 (4.79, 9.14)	6.30 (4.87, 8.69)	0.798	0.003
Cr, (umol/L)	73.00 (60.00, 93.65)	75.92 (63.60, 98.17)	0.007	0.013	73.90 (59.70, 94.92)	75.35 (63.60, 96.97)	0.140	0.015
CRP, (mg/L)	4.40 (1.04, 14.53)	8.38 (1.95, 33.23)	<0.001	0.324	6.20 (1.83, 24.16)	7.34 (1.71, 29.68)	0.576	0.033
WBC, (×10^9^/L)	2.29 (1.52, 3.85)	2.15 (1.44, 3.42)	0.018	0.168	2.09 (1.43, 3.54)	2.12 (1.42, 3.43)	0.803	0.058
Neutrophil, (×10^9^/L)	1.33 (0.80, 2.40)	1.19 (0.72, 2.10)	0.003	0.156	1.18 (0.73, 2.11)	1.19 (0.72, 2.15)	0.948	0.022
Lymphocyte, (×10^9^/L)	0.60 (0.40, 1.00)	0.52 (0.37, 0.82)	0.005	0.076	0.57 (0.38, 0.90)	0.52 (0.37, 0.81)	0.300	0.002
HGB, (g/L)	120.00 (104.00, 133.00)	115.00 (102.00, 129.00)	<0.001	0.145	116.00 (99.00, 129.75)	116.00 (103.00, 129.00)	0.602	0.051
PLT, (×10^9^/L)	48.00 (33.00, 69.00)	45.00 (30.00, 63.00)	0.001	0.182	45.00 (30.00, 66.00)	46.00 (31.00, 63.00)	0.815	0.042
PT, (s)	12.90 (12.00, 13.80)	13.60 (12.70, 14.60)	<0.001	0.087	13.22 (12.22, 14.20)	13.60 (12.60, 14.57)	<0.001	0.037
INR	1.00 (0.93, 1.08)	1.06 (0.98, 1.15)	<0.001	0.041	1.03 (0.95, 1.11)	1.05 (0.98, 1.14)	<0.001	0.009
APTT, (s)	49.00 (41.30, 58.30)	50.30 (42.48, 61.50)	0.006	0.152	49.90 (42.40, 59.68)	50.10 (42.32, 60.82)	0.696	0.005
FIB, (g/L)	2.69 (2.25, 3.28)	3.07 (2.53, 3.89)	<0.001	0.078	2.76 (2.29, 3.54)	3.04 (2.52, 3.85)	<0.001	0.042
D-D, (μg/L)	2.10 (1.13, 4.29)	2.73 (1.51, 6.26)	<0.001	0.026	2.26 (1.27, 4.85)	2.62 (1.40, 6.02)	0.056	0.069
Viral load (log10 copies/mL)	5.35 (4.61, 6.43)	5.17 (4.70, 6.24)	0.153	0.084	5.20 (4.56, 6.30)	5.11 (4.66, 6.24)	0.869	0.019

Abbreviations: SBP, systolic blood pressure; DBP, diastolic blood pressure; DM, diabetes mellitus; GLU, glucose; ALB, albumin; GLO, globulin; TBIL, total bilirubin; ALT, alanine aminotransferase; AST, aspartate aminotransferase; LDH, lactate dehydrogenase; BUN, blood urea nitrogen; Cr, creatinine; CRP, C-reactive protein; WBC, white blood cell; HGB, hemoglobin; PLT, platelet count; PT, prothrombin time; INR, international normalized ratio; APTT, activated partial thromboplastin time; FIB, fibrinogen; D-D, D-dimer; PSM, propensity score matching; SMD, standardized mean difference.

**Table 2:** Clinical and laboratory characteristics of the minocycline and control groups in the cytokine analysis cohort before and after PSM.

Characteristics	Control Group (*n* = 524)	Minocycline Group (*n* = 213)	*p*-Value	SMD	Control Group (*n* = 197)	Minocycline Group (*n* = 197)	*p*-Value	SMD
Basic characteristic								
Age, year	68.00 (58.00, 74.00)	65.00 (56.00, 73.00)	0.075	0.168	67.00 (56.00, 72.00)	65.00 (56.00, 74.00)	0.861	0.022
Male, *n* (%)	234 (44.7%)	112 (52.6%)	0.061	0.079	106 (53.8%)	102 (51.8%)	0.762	0.020
SBP, (mmHg)	112.50 (102.00, 126.25)	112.00 (99.00, 124.00)	0.271	0.102	113.00 (102.00, 126.00)	113.00 (101.00, 124.00)	0.808	0.027
DBP, (mmHg)	71.00 (63.00, 79.00)	70.00 (62.00, 78.00)	0.753	0.018	71.00 (64.00, 81.00)	71.00 (63.00, 78.00)	0.423	0.085
History								
DM, *n* (%)	91 (17.4%)	30 (14.1%)	0.327	0.033	25 (12.7%)	29 (14.7%)	0.660	0.020
Hypertension, *n* (%)	154 (29.4%)	62 (29.1%)	1.000	0.003	61 (31.0%)	57 (28.9%)	0.741	0.020
Laboratory parameters								
GLU, (mmol/L)	7.26 (5.84, 9.91)	7.45 (5.99, 10.27)	0.342	0.019	7.14 (5.71, 9.55)	7.43 (5.92, 10.27)	0.173	0.034
ALB, (g/L)	31.57 (5.63)	30.94 (6.24)	0.183	0.106	31.30 (6.19)	31.11 (6.23)	0.762	0.032
GLO, (g/L)	27.20 (24.10, 30.63)	26.20 (23.10, 28.70)	0.004	0.262	25.60 (22.90, 28.90)	26.50 (23.50, 29.20)	0.285	0.074
TBIL, (umol/L)	12.90 (8.36, 20.95)	14.50 (9.10, 23.60)	0.043	0.181	13.40 (8.80, 23.50)	14.10 (8.90, 22.42)	0.751	0.104
ALT, (U/L)	72.00 (41.00, 120.00)	70.00 (38.00, 141.00)	0.615	0.108	80.00 (38.00, 132.00)	67.00 (37.00, 138.00)	0.716	0.018
AST, (U/L)	141.00 (74.00, 282.30)	146.00 (73.00, 325.60)	0.724	0.091	155.00 (74.00, 339.00)	146.00 (73.00, 303.00)	0.570	0.028
LDH, (U/L)	593.00 (382.80, 1116.75)	665.00 (390.00, 1450.00)	0.100	0.211	635.00 (386.00, 1288.00)	663.00 (375.00, 1270.00)	0.904	0.003
BUN, (mmol/L)	6.75 (5.00, 10.00)	6.60 (5.00, 9.96)	0.899	0.128	7.38 (5.10, 10.57)	6.53 (5.00, 9.80)	0.218	0.072
Cr, (umol/L)	74.40 (61.02, 94.17)	78.80 (65.48, 103.50)	0.019	0.087	80.70 (64.00, 100.80)	78.30 (65.48, 102.10)	0.974	0.060
CRP, (mg/L)	5.05 (1.64, 15.03)	10.00 (2.12, 36.65)	<0.001	0.366	7.28 (2.29, 24.70)	7.82 (1.78, 31.17)	0.971	0.090
WBC, (×10^9^/L)	2.34 (1.60, 4.14)	2.23 (1.61, 3.35)	0.404	0.158	2.02 (1.50, 3.52)	2.23 (1.61, 3.35)	0.208	0.081
Neutrophil, (×10^9^/L)	1.37 (0.86, 2.55)	1.39 (0.90, 2.34)	0.714	0.097	1.29 (0.86, 2.16)	1.37 (0.90, 2.34)	0.675	0.074
Lymphocyte, (×10^9^/L)	0.54 (0.38, 0.97)	0.50 (0.35, 0.74)	0.024	0.089	0.50 (0.35, 0.75)	0.51 (0.36, 0.78)	0.786	0.005
HGB, (g/L)	120.00 (102.75, 133.00)	116.00 (101.00, 132.00)	0.181	0.128	119.00 (101.00, 131.00)	117.00 (103.00, 134.00)	0.968	0.008
PLT, (×10^9^/L)	47.50 (32.00, 70.00)	47.00 (33.00, 65.00)	0.898	0.070	45.00 (30.00, 69.00)	47.00 (33.00, 66.00)	0.652	0.001
PT, (s)	13.00 (11.90, 14.00)	13.50 (12.50, 14.60)	<0.001	0.069	13.20 (12.20, 14.20)	13.50 (12.50, 14.40)	0.117	0.007
INR	1.03 (0.95, 1.10)	1.06 (0.98, 1.15)	<0.001	0.053	1.04 (0.96, 1.12)	1.06 (0.98, 1.14)	0.195	0.003
APTT, (s)	48.75 (40.30, 58.47)	49.50 (42.50, 59.00)	0.124	0.065	49.20 (40.60, 61.60)	49.50 (42.50, 58.90)	0.652	0.017
FIB, (g/L)	2.72 (2.22, 3.34)	2.92 (2.43, 3.83)	<0.001	0.183	2.87 (2.34, 3.63)	2.89 (2.41, 3.65)	0.796	0.061
D-D, (μg/L)	2.01 (0.95, 4.03)	2.62 (1.56, 6.21)	0.001	0.010	2.57 (1.02, 5.41)	2.52 (1.43, 5.87)	0.438	0.027
Viral load (log10 copies/mL)	5.18 (4.70, 6.26)	5.41 (4.70, 6.57)	0.038	0.172	5.44 (4.70, 6.56)	5.39 (4.70, 6.53)	0.659	0.069

Abbreviations: SBP, systolic blood pressure; DBP, diastolic blood pressure; DM, diabetes mellitus; GLU, glucose; ALB, albumin; GLO, globulin; TBIL, total bilirubin; ALT, alanine aminotransferase; AST, aspartate aminotransferase; LDH, lactate dehydrogenase; BUN, blood urea nitrogen; Cr, creatinine; CRP, C-reactive protein; WBC, white blood cell; HGB, hemoglobin; PLT, platelet count; PT, prothrombin time; INR, international normalized ratio; APTT, activated partial thromboplastin time; FIB, fibrinogen; D-D, D-dimer; PSM, propensity score matching; SMD, standardized mean difference.

**Table 3:** Serum cytokine levels in the minocycline and control groups after PSM.

Cytokine (pg/mL)	Control Group (*n* = 197)	Minocycline Group (*n* = 197)	*p*-Value
IL-1β	3.53 (1.98, 8.69)	2.41 (2.13, 6.22)	0.898
IL-2	1.71 (1.14, 2.38)	1.66 (1.14, 2.16)	0.878
IL-4	1.58 (0.76, 1.94)	1.58 (0.78, 1.97)	0.512
IL-5	1.89 (1.19, 3.20)	1.89 (1.32, 2.81)	0.966
IL-6	16.30 (6.39, 59.83)	16.35 (5.16, 45.03)	0.495
IL-8	33.10 (12.30, 78.80)	21.63 (6.50, 48.64)	0.008
IL-10	13.83 (4.47, 59.97)	8.69 (3.22, 44.29)	0.035
IL-12p70	1.76 (1.43, 2.14)	1.73 (1.41, 2.04)	0.446
IL-17	2.01 (1.30, 3.12)	2.07 (1.47, 3.19)	0.952
TNF-α	3.40 (2.13, 15.09)	3.30 (2.21, 12.10)	0.809
IFN-α	18.99 (2.25, 98.50)	14.76 (2.42, 96.28)	0.949
IFN-γ	84.00 (24.74, 322.00)	86.33 (19.18, 278.56)	0.752

Abbreviations: IL-1β, interleukin-1 beta; IL-2, interleukin-2; IL-4, interleukin-4; IL-5, interleukin-5; IL-6, interleukin-6; IL-8, interleukin-8; IL-10, interleukin-10; IL-12p70, interleukin-12 p70; IL-17, interleukin-17; TNF-α, tumor necrosis factor-alpha; IFN-α, interferon-alpha; IFN-γ, interferon-gamma; PSM, propensity score matching.

## Data Availability

The raw data can be obtained by contacting the corresponding author.

## Supplementary Materials

The following supporting information can be downloaded at: https://www.mdpi.com/article/10.3390/pathogens15101032/s1. Figure S1. Covariate balance before and after propensity score matching. Standardized mean differences (SMDs) for all included covariates are presented. A dashed vertical line at SMD = 0.10 indicates acceptable balance. Abbreviations: SBP, systolic blood pressure; DBP, diastolic blood pressure; DM, diabetes mellitus; ALB, albumin; GLO, globulin; TBIL, total bilirubin; ALT, alanine aminotransferase; AST, aspartate aminotransferase; LDH, lactate dehydrogenase; BUN, blood urea nitrogen; Cr, creatinine; GLU, glucose; CRP, C-reactive protein; WBC, white blood cell; HGB, hemoglobin; PLT, platelet count; PT, prothrombin time; INR, international normalized ratio; APTT, activated partial thromboplastin time; FIB, fibrinogen; D-D, D-dimer; PSM, propensity score matching. Table S1. Characteristics of patients hospitalized for less than 3 days. Abbreviations: SBP, systolic blood pressure; DBP, diastolic blood pressure; DM, diabetes mellitus; GLU, glucose; ALB, albumin; GLO, globulin; TBIL, total bilirubin; ALT, alanine aminotransferase; AST, aspartate aminotransferase; LDH, lactate dehydrogenase; BUN, blood urea nitrogen; Cr, creatinine; CRP, C-reactive protein; WBC, white blood cell; HGB, hemoglobin; PLT, platelet count; PT, prothrombin time; INR, international normalized ratio; APTT, activated partial thromboplastin time; FIB, fibrinogen; D-D, D-dimer. Figure S2. Incidence of infection stratified by age in the control and minocycline groups. (A) Incidence of secondary infection in the minocycline and control groups. (B) Incidence of lung infection in the minocycline and control groups. (C) Incidence of urinary tract infection in the minocycline and control groups. (D) Incidence of bloodstream infection in the minocycline and control groups. Figure S3. Incidence of infection stratified by sex in the control and minocycline groups. (A) Incidence of secondary infection in the minocycline and control groups. (B) Incidence of lung infection in the minocycline and control groups. (C) Incidence of urinary tract infection in the minocycline and control groups. (D) Incidence of bloodstream infection in the minocycline and control groups. Figure S4. Incidence of infection stratified by SFTSV viral load in the control and minocycline groups. (A) Incidence of secondary infection in the minocycline and control groups. (B) Incidence of lung infection in the minocycline and control groups. (C) Incidence of urinary tract infection in the minocycline and control groups. (D) Incidence of bloodstream infection in the minocycline and control groups. Abbreviations: SFTSV, severe fever with thrombocytopenia syndrome virus. Figure S5. Incidence of infection stratified by diabetes mellitus status in the control and minocycline groups. (A) Incidence of secondary infection in the minocycline and control groups. (B) Incidence of lung infection in the minocycline and control groups. (C) Incidence of urinary tract infection in the minocycline and control groups. (D) Incidence of bloodstream infection in the minocycline and control groups. Abbreviations: DM, diabetes mellitus. Figure S6. Incidence of infection stratified by glucocorticoid use in the control and minocycline groups. (A) Incidence of secondary infection in the minocycline and control groups. (B) Incidence of lung infection in the minocycline and control groups. (C) Incidence of urinary tract infection in the minocycline and control groups. (D) Incidence of bloodstream infection in the minocycline and control groups. Figure S7. Incidence of infection stratified by IVIG use in the control and minocycline groups. (A) Incidence of secondary infection in the minocycline and control groups. (B) Incidence of lung infection in the minocycline and control groups. (C) Incidence of urinary tract infection in the minocycline and control groups. (D) Incidence of bloodstream infection in the minocycline and control groups. Abbreviations: IVIG, intravenous immunoglobulin. Table S2. Univariate logistic regression analysis of factors associated with combined infection in SFTS patients after propensity score matching. Abbreviations: ALB, albumin; TBIL, total bilirubin; ALT, alanine aminotransferase; AST, aspartate aminotransferase; LDH, lactate dehydrogenase; Cr, creatinine; GLU, glucose; CRP, C-reactive protein; WBC, white blood cell; HGB, hemoglobin; PLT, platelet count; PT, prothrombin time; INR, international normalized ratio; APTT, activated partial thromboplastin time; FIB, fibrinogen; D-D, D-dimer; IVIG, intravenous immunoglobulin. Figure S8. Covariate balance before and after propensity score matching. Standardized mean differences (SMDs) for all included covariates are presented. A dashed vertical line at SMD = 0.10 indicates acceptable balance. Abbreviations: SBP, systolic blood pressure; DBP, diastolic blood pressure; DM, diabetes mellitus; ALB, albumin; GLO, globulin; TBIL, total bilirubin; ALT, alanine aminotransferase; AST, aspartate aminotransferase; LDH, lactate dehydrogenase; BUN, blood urea nitrogen; Cr, creatinine; GLU, glucose; CRP, C-reactive protein; WBC, white blood cell; HGB, hemoglobin; PLT, platelet count; PT, prothrombin time; INR, international normalized ratio; APTT, activated partial thromboplastin time; FIB, fibrinogen; D-D, D-dimer.
