# Supplementary material for: Association of Early Minocycline Use with Secondary Infections in Patients with Severe Fever with Thrombocytopenia Syndrome: A Multicenter Retrospective Cohort Study
Source: Pathogens. 2026 Oct 1;15(10):1032. doi: 10.3390/pathogens15101032 (PMC13708476; doi:10.3390/pathogens15101032)
Supplement: Supplementary file 1 [file pathogens-15-01032-s001.zip › Table S1.pdf]

Table S1. Characteristics of patients hospitalized for less than 3 days

Characteristics	Hospital stay <3 days (n = 98)	Hospital stay≥3 days (n = 1975)	p-value
**Basic characteristic**			
Age, year	71.00 (64.00, 77.75)	67.00 (58.00, 73.00)	<0.001
Male, n (%)	47 (48.0%)	861 (43.6%)	0.456
SBP, (mmHg)	114.50 (100.25, 132.75)	114.00 (102.00, 126.00)	0.262
DBP, (mmHg)	71.00 (64.00, 83.75)	71.00 (64.00, 80.00)	0.622
**History**			
DM, n (%)	8 (8.2%)	256 (13.0%)	0.217
Hypertension, n (%)	32 (32.7%)	579 (29.3%)	0.553
**Laboratory parameters**			
GLU, (mmol/L)	7.94 (6.20, 11.14)	6.96 (5.71, 9.48)	0.005
ALB, (g/L)	30.75 (26.52, 34.70)	31.80 (27.70, 36.00)	0.109
GLO, (g/L)	27.20 (24.13, 32.00)	26.30 (23.30, 29.60)	0.020
TBIL, (umol/L)	11.02 (8.23, 17.45)	12.30 (8.57, 20.40)	0.110
ALT, (U/L)	95.00 (63.50, 170.25)	76.00 (43.00, 136.50)	0.002
AST, (U/L)	405.40 (179.75, 705.00)	145.00 (76.00, 300.50)	<0.001
LDH, (U/L)	1318.60 (701.00, 3643.00)	625.00 (377.00, 1201.00)	<0.001
BUN, (mmol/L)	9.70 (6.23, 14.85)	6.50 (4.91, 9.13)	<0.001
Cr, (umol/L)	100.80 (69.62, 200.25)	73.40 (61.00, 94.75)	<0.001
CRP, (mg/L)	6.69 (1.57, 17.90)	5.03 (1.20, 17.74)	0.252
WBC, ($\times 10^9$ /L)	2.92 (1.72, 5.14)	2.23 (1.50, 3.71)	0.004
Neutrophil, ($\times 10^9$ /L)	1.81 (0.99, 3.44)	1.29 (0.78, 2.31)	0.001
Lymphocyte, ($\times 10^9$ /L)	0.62 (0.38, 1.08)	0.58 (0.39, 0.95)	0.273
HGB, (g/L)	129.00 (120.25, 143.50)	119.00 (103.00, 132.00)	<0.001
PLT, ($\times 10^9$ /L)	40.50 (25.00, 53.75)	48.00 (33.00, 67.00)	0.004
PT, (sec)	13.70 (13.00, 15.00)	13.10 (12.20, 14.10)	<0.001
INR	1.04 (0.98, 1.17)	1.02 (0.94, 1.10)	0.001
APTT, (sec)	67.30 (50.15, 95.58)	49.40 (41.60, 59.10)	<0.001
FIB, (g/L)	2.33 (1.87, 2.80)	2.80 (2.31, 3.43)	<0.001
D-D, (μg/L)	5.60 (2.64, 10.57)	2.24 (1.20, 4.80)	<0.001
Viral load (log10 copies/mL)	6.46 (5.36, 7.91)	5.30 (4.66, 6.37)	<0.001

Abbreviations: SBP, systolic blood pressure; DBP, diastolic blood pressure; DM, diabetes mellitus; GLU, glucose; ALB, albumin; GLO, globulin; TBIL, total bilirubin; ALT, alanine aminotransferase; AST, aspartate aminotransferase; LDH, lactate dehydrogenase; BUN, blood urea nitrogen; Cr, creatinine; CRP, C-reactive protein; WBC, white blood cell; HGB, hemoglobin; PLT, platelet count; PT, pro-thrombin time; INR, international normalized ratio; APTT, activated partial thromboplastin time; FIB, fibrinogen; D-D, D-dimer.
