# Supplementary material for: Association of Early Minocycline Use with Secondary Infections in Patients with Severe Fever with Thrombocytopenia Syndrome: A Multicenter Retrospective Cohort Study
Source: Pathogens. 2026 Oct 1;15(10):1032. doi: 10.3390/pathogens15101032 (PMC13708476; doi:10.3390/pathogens15101032)
Supplement: Supplementary file 1 [file pathogens-15-01032-s001.zip › Table S2.pdf]

Table S2. Univariate logistic regression analysis of factors associated with combined infection in SFTS patients after propensity score matching.

Variable	Univariable		
	OR	95% CI	P-value
Minocycline use	0.70	0.55 - 0.90	0.006
Disease security	2.89	2.16 - 3.86	<0.001
Age	1.05	1.04 - 1.06	0.000
Sex	0.89	0.70 - 1.14	0.368
ALB	0.90	0.88 - 0.92	0.000
TBIL	1.01	1.01 - 1.02	0.000
ALT	1.00	1.00 - 1.00	0.116
AST	1.00	1.00 - 1.00	0.001
LDH	1.00	1.00 - 1.00	0.002
Cr	1.01	1.00 - 1.01	0.000
Glu	1.11	1.08 - 1.16	0.000
CRP	1.01	1.01 - 1.02	0.000
WBC	1.01	0.95 - 1.07	0.770
Neutrophil	1.05	0.98 - 1.13	0.138
Lymphocyte	0.66	0.51 - 0.86	0.002
HGB	0.98	0.98 - 0.99	0.000
PLT	0.99	0.99 - 0.99	0.000
PT	1.02	0.99 - 1.05	0.241
APTT	1.01	1.00 - 1.01	0.002
FIB	1.00	0.99 - 1.02	0.903
D-D	1.00	0.99 - 1.00	0.512
Viral load(log 10)	1.18	1.09 - 1.27	0.000
IVIG use	2.19	1.67 - 2.88	0.000
Glucocorticoids use	1.68	1.25 - 2.25	0.001

Abbreviations: ALB, albumin; TBIL, total bilirubin; ALT, alanine aminotransferase; AST, aspartate aminotransferase; LDH, lactate dehydrogenase; Cr, creatinine; GLU, glucose; CRP, C-reactive protein; WBC, white blood cell; HGB, hemoglobin; PLT, platelet count; PT, prothrombin time; INR, international normalized ratio; APTT, activated partial thromboplastin time; FIB, fibrinogen; D-D, D-dimer; IVIG, intravenous immunoglobulin.
